# Levetiracetam-Induced Acute Psychosis in an Adolescent

**DOI:** 10.1155/2023/5575900

**Published:** 2023-09-15

**Authors:** Omkar Dhungel, Amit Shrestha, Pankaj Pathak, Pawan Sharma

**Affiliations:** Patan Academy of Health Sciences, School of Medicine, Department of Psychiatry, Lalitpur, Nepal

## Abstract

Levetiracetam (LEV), a second-generation antiepileptic, is used as an adjunct therapy in primary generalized tonic–clonic seizures, refractory partial-onset seizures, and seizure prophylaxis after brain surgery. It is well tolerated, effective and has a convenient dosing regimen. As any other drugs, it has some adverse drug effects, including neuropsychiatric adverse effects ranging from agitation and mood symptoms to psychosis and suicide. Strong diagnostics guidelines are yet to be formulated for LEV-induced psychosis; however, complete recovery from psychotic symptoms after stopping LEV supports the possible adverse reaction from Naranjo's algorithm and, hence, the diagnosis. This case report presents a 16 years boy with focal onset generalized tonic–clonic seizure, whose drug regimen was switched to LEV, following which he had the delusion of persecution, second-person auditory hallucination, and aggressive behavior, which decreased on the 2nd day of cessation of LEV.

## 1. Introduction

Levetiracetam (LEV) is an effective adjunctive therapy for refractory partial-onset seizures, primary generalized tonic–clonic seizures, myoclonic seizures of juvenile myoclonic epilepsy, and post-brain surgery seizure prophylaxis [1]. LEV was approved in 1999 by the United States Food and Drug Administration (FDA) as an adjuvant antiepileptic medication for partial seizures in adults. In long-term, open-labeled, follow-up studies, LEV demonstrated good seizure control [2]. The proposed mechanism of action involves binding to synaptic vesicle protein 2A, which leads to neuronal inhibition [3]. It also enhances the concentration of GABA by its interaction with the GABA-A receptor in the brain, decreases glutamatergic excitation by modulation of the *N*-methyl-D-aspartate receptor and the *α*-amino-3-hydroxy-5-methyl 4-isoxazole-propionic receptors and upregulation of glial glutamate transporters leading to neuroprotective action [4]. LEV is minimally bound with protein and undergoes minimal metabolism by the cytochrome P450 system. It has a convenient dosing regimen and a wide therapeutic index, which does not necessitate strict serum drug monitoring [5].

The iatrogenic, adverse drug reaction (ADR) caused by antiepileptic is termed as antiepileptic drug (AED)-induced psychotic disorder (AIPD). AIPD was common in younger patients with focal onset seizures [6]. In clinical trials of AEDs, the prevalence of AIPD was found in the range of 1.0% to 8.4% [7]. Similarly, some people treated with LEV can experience ADRs. Approximately 18% of people treated with LEV for epilepsy may experience some neuropsychiatric symptoms, which need a decrease in dosage or cessation of LEV treatment [8]. Among different neuropsychiatric adverse effects, psychosis has been reported infrequently with LEV, with a reported frequency of less than 1% [9]. The mechanism underlying the LEV-induced psychotic symptoms is still a subject of research. Psychotic symptoms during LEV therapy were significantly associated with status epilepticus, a history of psychotic symptoms, a history of psychiatric illness other than psychosis, female gender, a history of febrile convulsions, and intellectual disability [6]. Here, we present a case of 16 years old male with no history of psychiatric illness, whose frequent seizure episodes were remarkably under control with LEV treatment but manifested with psychotic symptoms on the 4th day, which disappeared within 2 days after LEV was cross-tapered to another AED.

## 2. Case History

A 16-year-old boy with no history of psychiatric illness in self or family and no psychoactive substance use presented with a seizure disorder of 5 years' duration. The semiology of the seizure was such that there would be an aura-tingling sensation in limbs, ringing in ears, and fearfulness for less than a minute, followed by focal onset seizure-twisting of the upper limb and head toward left with stiffening and jerky movements of all four limbs with unconsciousness associated with tongue bite, injury due to fall and episodes also occurred during sleep. He would be confused for a few minutes after gaining consciousness, complain of headaches, and would sleep at times. He had stopped going to school for 4 years and would refuse to visit his relatives because of fear of a seizure episode. He was treated with sodium valproate up to 800 mg for a year, 2 years back with irregular follow-ups. As a few episodes of seizure occurred in-between, the family opted for treatment from traditional healers instead of medicine, and he had been noncompliant for a year. For the last 4 months, the frequency had increased to 1–3 episodes within a day.

A detailed clinical evaluation was done, and he was diagnosed as a case of focal onset generalized tonic–clonic seizure and advised for baseline blood investigations, EEG, and MRI and was restarted on sodium valproate 300 mg BD with the view of using an adequate dose and optimized to 900 mg in a week. The general physical and systemic examination, blood parameters, and MRI brain were normal. EEG showed slow background activity with sharp and spike-wave complexes in both cerebral hemispheres.

**Figure 1:**
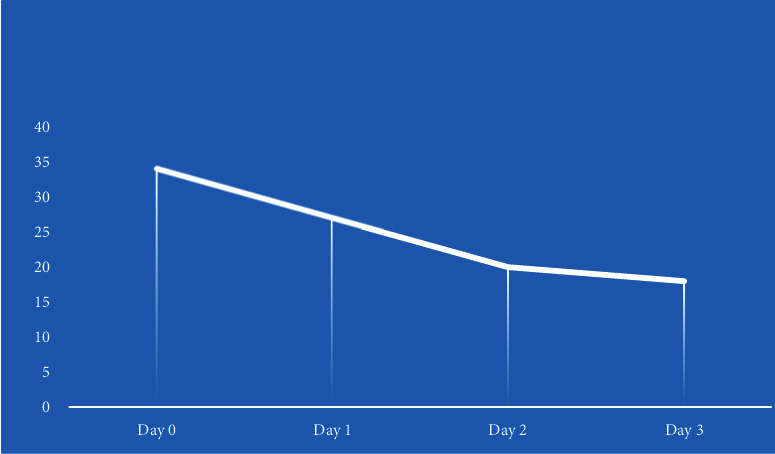
Change in BPRS score.

**Figure 2:**
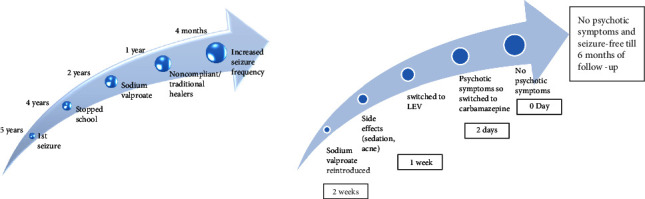
Timeline of illness.

After optimizing the dose of valproate, he was seizure-free for 6 days. However, he presented to the emergency department with drowsiness, decreased alertness, lethargy, and drooling of saliva with a serum valproate level of 76 mg/L. In view of the sedating side effect of sodium valproate, the antiepileptic was changed to tablet LEV 500 mg BD. The drowsiness improved after stopping valproate and starting LEV. On the 2nd day, he started saying that he felt like going crazy. On the fourth day, he was restless, agitated, and tried to run away from the ward with delusion of persecution and auditory hallucination, second person voices commenting. The brief psychiatric rating scale (BPRS) score was 34 (Figure 1), with scores in suspiciousness, hostility, and uncooperativeness. He was seen by a consultant psychiatrist, and a provisional diagnosis of LEV-induced psychosis with epilepsy was made, with a score of 4 in the Naranjo adverse drug scale, which signifies the possibility of ADR. Though the serum LEV level could not be assessed due to unavailability, in view of psychotic symptoms following LEV introduction, it was switched to tablet carbamazepine 200 mg HS. There was an improvement in agitation and psychotic symptoms within 2 days of stopping LEV; the BPRS score was 27 and 18 (Figure 1) subsequently, and he was discharged 2 days later without any antipsychotic medications. The dosage of carbamazepine was optimized to 800 mg, and there was no new episode of seizure or psychotic symptoms till 6 months of follow-up (Figure 2).

## 3. Discussion

LEV is an effective AED with a good safety profile, but some patients treated with LEV experience neuropsychiatric ADRs. It was seen that approximately 18% of people treated with LEV for epilepsy experienced some neuropsychiatric symptoms with psychotic symptoms in less than 1%, which forced to decrease the dosage or cessation of LEV treatment [8, 9]. Some predisposing factors are history of psychosis, secondary generalized seizures, absence seizures, and intractable epilepsy [8]. There are no agreed definitions or diagnostic criteria for AIPD in the classification systems of either epileptology or psychiatry. When the offending agents were AEDs, the disorder was classified as AIPD with specific diagnostics criteria [9].

An open-label, noncomparative, multicenter, long-term follow-up study done by Delanty et al. showed that despite a good safety profile, LEV led to psychiatric side effects in up to 13.3% of adults and 37.6% of pediatric patients. Among them, significant symptoms such as depression, agitation/hostility, and psychotic behavior were observed in 0.7% of patients [10]. A reported case of 52-year-old males with epilepsy who developed acute psychosis shortly after initiation of treatment. Within 2 days of LEV discontinuation, the patient recovered from psychosis without any treatment [11]. A systematic review by Cramer [12] demonstrated that LEV-induced psychiatric symptoms were reversible following withdrawal of the medication. A prevalence study done by Piedad et al. [7] demonstrated using logistic regression analysis that the use of carbamazepine was negatively associated with AIPD, compared with other types of psychosis.

The possibility of postictal psychosis is minimal according to Logsdail and Toone's diagnostic criteria [13], as he was seizure-free for around 10 days with the antiepileptic. In our patient, there were no prior psychiatric illness or psychoactive substance use with normal physical and lab parameters, including MRI with temporal relationship between the initiation of LEV and psychotic symptoms and improvement in symptoms after stopping LEV and starting carbamazepine. These all evidence and also, according to Naranjo et al. [14] Adverse drug scale, there is a possible relationship between LEV and psychosis in our patient.

The limitation of our study was that we could not analyze the serum LEV level, which could have increased the score in the Naranjo algorithm, but the studies have clearly mentioned that the LEV-induced psychotic symptoms are not dose-dependent.

In conclusion, though a safe molecule, LEV has the propensity to induce psychosis, for which we should be vigilant of the risk factors as mentioned. Close clinical monitoring of psychiatric adverse effects is expected from every clinician when starting treatment with LEV. Early identification and drug modification can reduce the morbidity and burden to the patient, family, and the healthcare system. In addition, further large-scale studies are needed to assess the behavioral profile of LEV and identify the risk factors for LEV-induced psychosis.
